# Are parrots poor at motor self-regulation or is the cylinder task poor at measuring it?

**DOI:** 10.1007/s10071-017-1131-5

**Published:** 2017-09-19

**Authors:** Can Kabadayi, Anastasia Krasheninnikova, Laurie O’Neill, Joost van de Weijer, Mathias Osvath, Auguste M. P. von Bayern

**Affiliations:** 1 Department of Cognitive Science, Lund University, Helgonavägen 3, 22100 Lund, Sweden; 2 Max-Planck-Institute for Ornithology, Eberhard-Gwinner-str., 82319 Seewiesen, Germany; 3 Max-Planck Comparative Cognition Research Station, Loro Parque Fundacíon, 38400 Puerto de la Cruz, Tenerife Spain; 4 Centre for Languages and Literature, Lund University, Helgonabacken 12, 22362 Lund, Sweden

**Keywords:** Motor self-regulation, Inhibition, Detour-reaching task, Cylinder task, Self-control, Brain size, Psittacidae

## Abstract

The ability to inhibit unproductive motor responses triggered by salient stimuli is a fundamental inhibitory skill. Such motor self-regulation is thought to underlie more complex cognitive mechanisms, like self-control. Recently, a large-scale study, comparing 36 species, found that absolute brain size best predicted competence in motor inhibition, with great apes as the best performers. This was challenged when three *Corvus* species (corvids) were found to parallel great apes despite having much smaller absolute brain sizes. However, new analyses suggest that it is the number of pallial neurons, and not absolute brain size per se, that correlates with levels of motor inhibition. Both studies used the cylinder task, a detour-reaching test where food is presented behind a transparent barrier. We tested four species from the order Psittaciformes (parrots) on this task. Like corvids, many parrots have relatively large brains, high numbers of pallial neurons, and solve challenging cognitive tasks. Nonetheless, parrots performed markedly worse than the *Corvus* species in the cylinder task and exhibited strong learning effects in performance and response times. Our results suggest either that parrots are poor at controlling their motor impulses, and hence that pallial neuronal numbers do not always correlate with such skills, or that the widely used cylinder task may not be a good measure of motor inhibition.

**Electronic supplementary material:**

The online version of this article (doi:10.1007/s10071-017-1131-5) contains supplementary material, which is available to authorized users.

## Introduction

Inhibitory control—a core component of executive functions—operates at a range of levels from basic motor self-regulation to taxing self-control (Diamond 2013; Beran 2015). Whereas motor self-regulation requires only the suppressing of an unproductive movement, self-control involves the ability to decline an immediate, small reward in favor of a larger but delayed one. Great apes, corvids, and parrots are proficient in such self-control tasks and surpass most other tested species when it comes to the duration of the delays that are tolerated (e.g., Rosati et al. 2007; Dufour et al. 2012; Auersperg et al. 2013; Koepke et al. 2015). Recently, it has been shown that great apes and corvids of the *Corvus* genus also outperform most other species in motor self-regulation (MacLean et al. 2014; Kabadayi et al. 2016). The correlation in performance between self-control and motor self-regulation might hint at a common underlying executive function relating to brain capacity and overall cognitive flexibility. Indeed, MacLean and colleagues found that the best performers on motor self-regulation among 36 tested species were those with the largest absolute brain sizes (MacLean et al. 2014). Notably, however, this study included mostly primates and other mammals, and very few bird species; when more birds were tested in a subsequent study, it was shown that absolute brain size may not be the best predictor across phylogenetically distant taxa (Kabadayi et al. 2016; for discussion, see also Chappell 2016). Instead, when the results from both studies were reanalyzed, the success rates roughly correlated with total numbers of pallial neurons, as birds have vastly higher neuronal densities than mammals (Herculano-Houzel 2017; Olkowicz et al. 2016).

In each of these studies, the main test used for investigating motor self-regulation was the so-called *cylinder task*. It is a detour-reaching task where a reward is placed in the center of a transparent cylinder with openings at both ends. Subjects must inhibit their initial response to reach directly for the visible food through the transparent barrier and instead retrieve the food through one of the side openings. Before being tested, subjects are familiarized with the affordances of the task; they are exposed first to an opaque cylinder and must show they can reliably retrieve the reward from either of the side openings. It is also crucial that the subjects are familiar with transparent surfaces, having experienced that they act as barriers as opaque objects do. A failure in the tests following the familiarization with opaque cylinders is scored if the subject attempts to reach for the reward directly by bumping into the transparent barrier. Failure signifies the subject cannot inhibit the direct motor movement toward the visible reward despite having previously learnt the correct detour response by means of the opaque cylinder. Previous studies employing the cylinder paradigm have used performance across ten trials as a comparative measure of basic motor inhibition.

In order to examine whether total numbers of pallial neurons (Herculano-Houzel 2017) and high performance in previous cognitive studies (Güntürkün and Bugnyar 2016) indeed link to such basic functions as motor self-regulation, we tested four species of large parrots in the cylinder task. Parrots have brains as large, or larger than *Corvus* species and exhibit similarly high densities of pallial neurons (Olkowicz et al. 2016; Iwaniuk et al. 2004, 2005; Iwaniuk and Nelson 2003; Herculano-Houzel 2017). Several parrot species are also known to perform on par with apes and corvids in various tests on physical and social cognition (Auersperg et al. 2014; Schloegl et al. 2012; O’Hara et al. 2015; Pepperberg, 2009; Güntürkün and Bugnyar 2016). Hence, we predicted that if pallial neuron count and cognitive performance were linked with motor self-regulation ability, parrots should show similar performance to corvids and apes.

We tested four species of parrots in the cylinder task: blue-headed macaws (*Primolius couloni*), blue-throated macaws (*Ara glaucogularis*), great green macaws (*Ara ambiguus*), and African grey parrots (*Psittacus erithacus*). We followed the methods of the two previously mentioned studies (MacLean et al. 2014; Kabadayi et al. 2016).

## Methods

### Subjects

A total of 38 parrots participated in this study: Eight African grey parrots (six females, two males, all 1 year old), eight blue-headed macaws (five females, three males, all 1 year old), 13 blue-throated macaws (one female, twelve males, mean age 2.46, SD = 1.76), and nine great green macaws (eight females, one male, mean age 2.33, SD = 2.69). All parrots were hand-raised and subsequently socialized in parrot groups in the Loro Parque Fundacíon, Tenerife, Spain.

### Housing conditions

All parrots were housed in aviaries at the Max-Planck Comparative Cognition Research Station in the Loro Parque in Puerto de la Cruz, Tenerife. The blue-throated macaws and the great green macaws were housed in eight aviaries, divided by species and age into five groups of two to eight individuals.

Six of these aviaries were 1.80 × 3.40 × 3 m (width × length × height), and the remaining aviaries were 2 × 3.40 × 3 m and 1.5 × 3.40 × 3 m, respectively. These aviaries were interconnected by 1 m × 1 m windows, which could be closed when desired. The blue-headed macaws were housed together in a separate indoor area (28.61 m^2^) with access to a smaller outdoor area and the African grey parrots were housed together in another separate outdoor aviary (21.41 m^2^). All aviaries had at least one side open to the outside, so they followed a natural light schedule and were also kept to ambient outdoor temperature, but they were additionally lit with Arcadia Zoo Bars (Arcadia 54W Freshwater Pro and Arcadia 54W D3 Reptile lamp) to ensure sufficient exposure to UV light. They were also all within the same building as the testing chambers (described below).

### Experimental setup and procedures

Training and testing took place in an indoor chamber of 1.5 × 1.5 × 1.5 m (height × width × length) equipped with lamps covering the birds’ full range of visible light (Arcadia 39 W Freshwater Pro and Arcadia 39 W D3 Reptile lamp). The birds were already habituated to moving from aviaries to the testing chambers. The subjects were individually tested in one of the testing chambers with the experimenter in an adjoining room. A sound-buffered one-way glass system permitted zoo visitors to see inside the rooms, but did not allow the birds to see out. All training and testing sessions took place either in the morning or in the afternoon, a minimum of 4 h after the last feeding (or overnight for morning sessions). All birds had free access to water and mineral blocks at all times and were fed fresh fruit and vegetables twice a day. Pieces of walnuts were used as rewards during testing as they are valued by all individuals and were not available outside of testing. The daily amount of nuts and seeds provided to the birds was adjusted according to their intake during testing for weight regulation purposes.

**Fig. 1:**
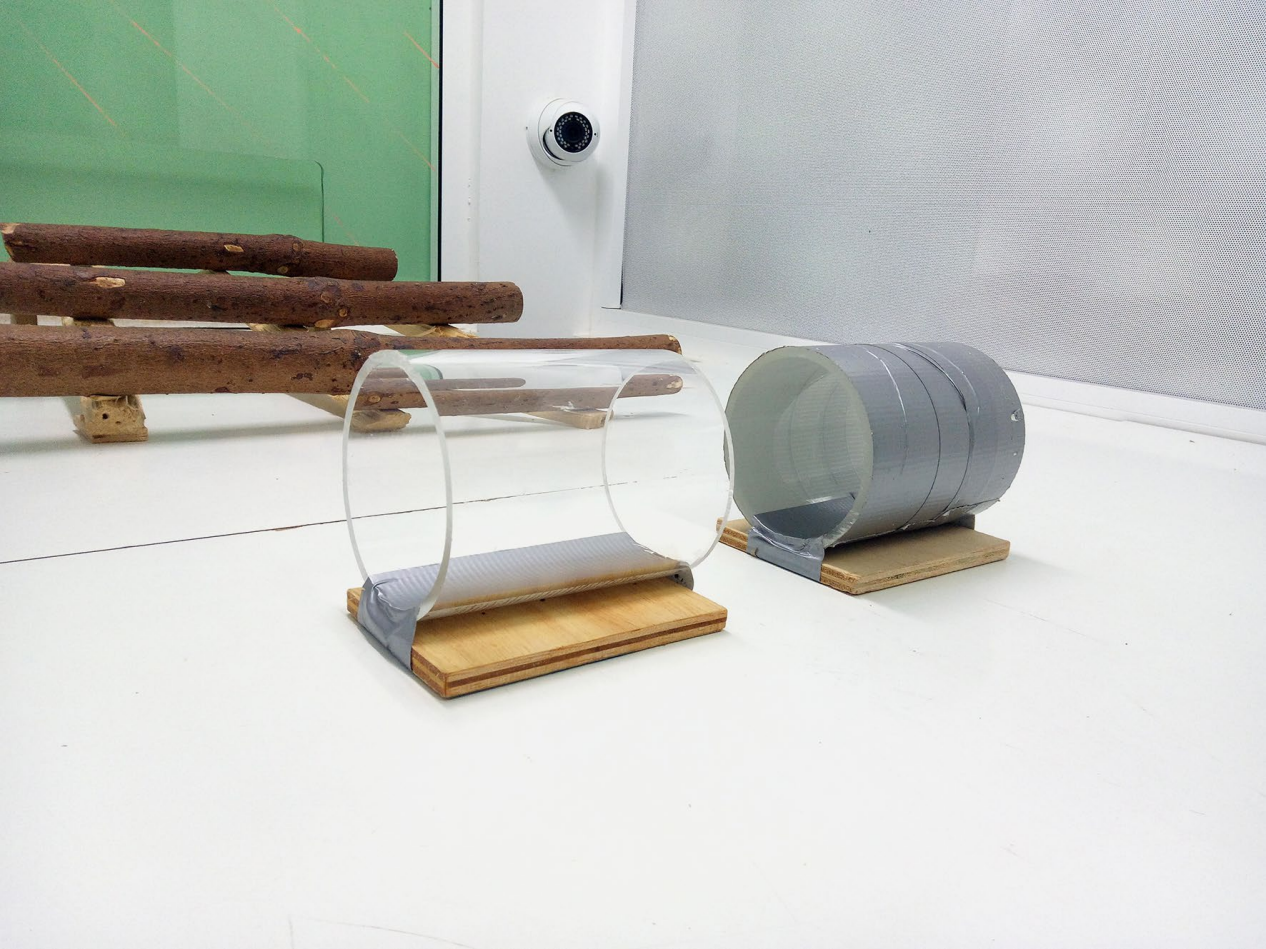
Cylinders used in the study (left: transparent cylinder, right: opaque cylinder)

The apparatus consisted of an opaque and a transparent cylinder (Fig. 1). Both cylinders were open at both ends and attached to a wooden base. Following the criterion set by MacLean et al. (2014), the cylinders were long enough so that the birds had to put their heads inside the cylinder to reach the reward in the center, but were not so large they could enter the cylinder entirely. The size of the cylinders was adjusted to the size of each species tested (a length of 15 cm and a diameter of 11 cm for the great green macaws, a length of 12.5 cm and a diameter of 9 cm for the blue-throated macaws, and a length of 10 cm and a diameter of 5 cm for the African grey parrots and the blue-headed macaws). For each species, the size of the opaque and transparent cylinder was identical. Prior to this study, the birds had participated in an extensive physical cognition test battery following the protocol of Herrmann et al. (2007), in which they interacted with humans through holes in a Plexiglas panel on a daily basis for 2 months, and consequently all subjects had experience with transparent surfaces. Although none of the tasks required an active contact with the Plexiglass panel, the birds did explore the panel by touching it with their beak and/or tongue in the course of testing.

### Training

In the training phase, the birds learned to retrieve a reward from either side opening of an opaque cylinder. Before each trial, the birds were given a signal (which they had been previously trained on) to wait on a perch at the back of the testing chamber. The experimenter then drew the bird’s attention to the reward (a piece of walnut) by holding it up at eye level and calling their name. The experimenter then placed the food inside the cylinder while the bird was observing, at which point the bird could approach the cylinder. Birds had 360° access to the cylinder. If the bird did not approach the cylinder within 2 min, the reward was removed from the cylinder, and the trial was repeated after a 30-s time-out interval. Hence, such invalid trials did not count toward the maximum of 10 trials per session. Correct responses were scored when the birds retrieved the food without touching the surface of the cylinder, whereas incorrect responses were scored when the birds made contact with the surface of the cylinder. The birds were allowed to retrieve the food after both correct and incorrect responses. When the parrots finished eating the reward, they were again given a signal to return to their perch, and the next trial commenced. To proceed to the testing stage, the birds had to fulfill a criterion of four out of five correct responses on consecutive trials following the criteria from MacLean et al. (2014) and Kabadayi et al. (2016). Birds were given a maximum of 10 trials per session. All birds reached criterion within three sessions, but the majority reached it on their first session.

### Testing

The testing protocol remained identical to the training procedure, with the exception that the opaque cylinder was now replaced with a transparent cylinder. Ten trials were conducted for each individual, except for two African grey parrots, who participated in one and two trials less, respectively, due to experimenter error. To preclude loss of motivation, the ten trials were divided into two sessions of five trials each, carried out on subsequent days. As in training, a correct response was coded if the birds made a detour to either side of the cylinder and retrieved the food without touching the surface of the cylinder, whereas an incorrect response was coded if the bird made physical contact with the surface of the cylinder before retrieving the food (Online Resource 1). All trials continued until the subject retrieved the reward. The methodology described above followed the one of MacLean et al. (2014) and Kabadayi et al. (2016) for both the training and the testing phase.

For all trials (correct and incorrect), we also measured the duration of time necessary for the birds to obtain the reward from the onset of the trial (response times). The onset of the trial was defined as the moment when the bird crossed a certain boundary line marked on the ground. The change in response times across trials was used in previous detour studies to study learning processes (Lockman and Adams 2001; Wyrwicka 1959). Thus, we analyzed the change in response times across trials *within* species as well as the difference in the rate of this change between species. Because of the slight between-species differences in distance between the mark on the ground and the cylinder, we did not compare response times between species.

### Analyses

Two variables were analyzed: the number of correct responses (response accuracy) and the response times per individual per trial. To analyze response accuracy, we used a generalized linear mixed-effect regression analysis (GLMM) with trial number, species, and bird age (in years) as fixed effects. Individual birds were included in the models as random effects, and the trial effect was allowed to vary for each individual bird (random slopes). The outcome variable was binary, i.e., the response was either correct or incorrect. To analyze response times, we used a linear mixed effects regression analysis (LMM) with the same fixed and random effects as in the analysis of the response accuracy. The outcome variable was response times in seconds.

### Failure patterns

We followed the coding criterion from the previous studies (MacLean et al. 2014; Kabadayi et al. 2016; Vernouillet et al. 2016), where all touches of the surface of the cylinder counted as an error, regardless of the location of the touches. However, we observed differences within the errors as some touches did not appear to be directed toward the reward, but could have been the result of exploration or accident. We therefore also provide additional analyses of the patterns of failures in order to potentially differentiate between failures caused by motor self-regulation and those caused by other factors.

In this additional analysis, we coded whether the parrots first touched the cylinder *toward* or *away* from the reward. When coding this, the cylinder was divided (on the computer screen) into three equal cross-sections (left periphery, center, right periphery), one of which contained the reward. If the birds’ initial contact with the cylinder was within the same zone as the reward then it was coded as a reach “toward” the reward; otherwise, it was coded as “away” from the reward. The interobserver reliability when coding for the failure patterns was excellent: Cohen’s Kappa = 0.961 (*n* = 135, *z* = 11.2, *p* < 0.001). We then recalculated the scores for all trials, with the following coding criterion: A correct response was coded if the birds made a detour to either side of the cylinder and retrieved the reward without touching the cylinder “toward” the reward, whereas an incorrect response was coded if the bird touched the cylinder “toward” the reward before retrieving the food. We then reran our original model on response accuracy using these new scores. Additionally, we analyzed how many errors were coded as either a reach toward or away from the reward across trials. We analyzed this using a generalized mixed model regression analysis, with error type as the outcome variable (i.e., toward the reward or away from reward), and species, trial number, and age as fixed effects. As in the first analysis, individual birds were included as random effects, and the trial effect was allowed to vary for each individual bird. All statistical analyses were carried in R, version 3.1.3 (R core team 2015).

## Results

The proportion of correct responses (response accuracy) for each of the four species is shown in Table 1. Scores ranged from 33% (blue-headed macaw) to 59% (great green macaw). There was an overall species effect on the scores (*χ* ^2^(2) = 8.707, *p* = 0.013). Great green macaws and blue-throated macaws significantly outperformed African grey parrots and blue-headed macaws (GLMM: EST = − 0.977, SE = 0.318, *z* = − 3.070, *p* = 0.002). However, there were no significant differences between the scores of great green macaws and blue-throated macaws (GLMM: EST = − 0.344, SE = 0.408, *z* = − 0.844, *p* = 0.399) and between African grey parrots and blue-headed macaws (GLMM: EST = 0.049, SE = 0.484, *z* = 0.103, *p* = 0.918).

**Table 1:** Species averages for the cylinder task scores using the original coding criterion (all touches were coded as a failure) and the new coding criterion (failures were coded only if the touch was directed toward the reward)

	Proportion correct	Proportion correct (new coding criterion)
African grey parrot	.34 (.19–.49)	.35 (.20–.50)
Blue-headed macaw	.33 (.18–.47)	.36 (.22–.51)
Blue-throated macaw	.51 (.38–.64)	.67 (.56–.79)
Great green macaw	.59 (.44–.74)	.77 (.64–.88)
Total (all species)	.45 (.36 –.53)	.57 (.47–.68)

Values in parentheses are the lower and upper limits of 95% confidence intervals

**Fig. 2:**
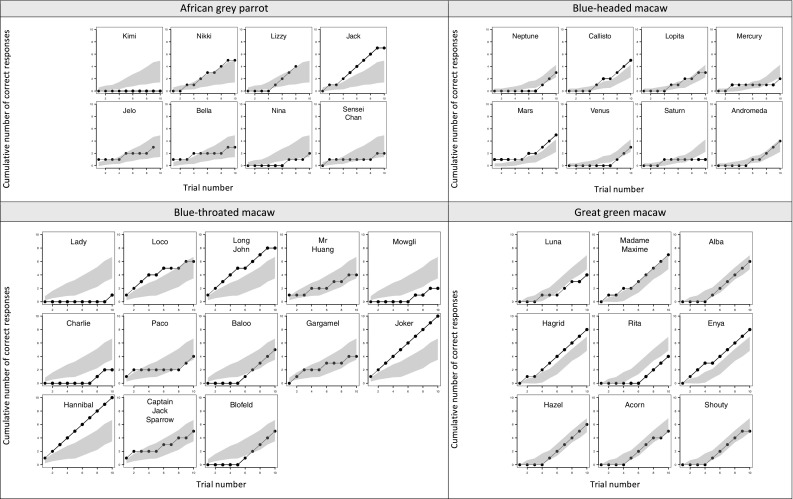
Individual learning curves of the 38 birds included in the study, presented separately for each species. Horizontal axes represent the trial numbers, and vertical axes represent the cumulative numbers of correct responses on the cylinder task. The black lines show the individual birds’ scores. The shaded gray areas indicate the upper and lower limits of 95% confidence intervals for the species averages

**Fig. 3:**
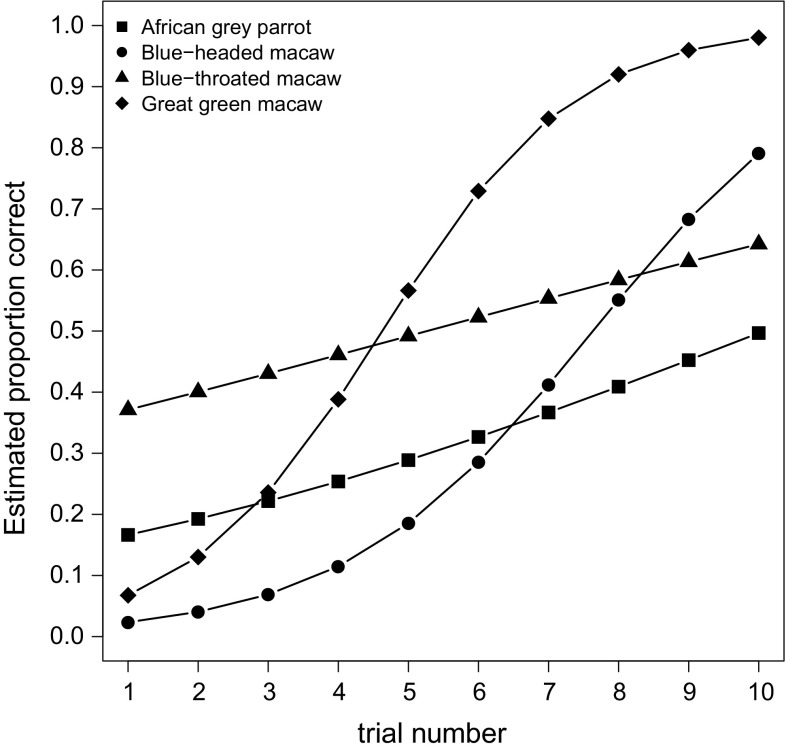
Each species’ estimated change in the cylinder task score (proportion correct responses) over the 10 trials, predicted from the generalized linear mixed-effect regression analysis, with correct or incorrect as the binary outcome variable

Analysis of response accuracy showed a significant effect of trial number across the four species (GLMM: EST = 3.587, SE = 0.651, *z* = 5.510, *p* < 0.001), suggesting that, as a group, the parrots improved their performance over trials (see Fig. 2 for individual/species performance across trials). Species also differed significantly in how much they improved their performance over trials, as evidenced by the interaction between trial and species (*χ* ^2^(3) = 13.827, *p* = 0.003; Fig. 3). The slopes of the curves of the two fastest learning species, the blue-headed macaws and the great green macaws, did not differ significantly from one another (GLMM: EST = − 1.510, SE = 1.927, *z* = − 0.783, *p* = 0.433), nor did the slopes of the curves for the two slowest learning species (i.e., the African grey parrots and the blue-throated macaws) differ significantly from each other (GLMM: EST = 0.480, SE = 1.431, *z* = 0.335, *p* = 0.738). However, the combined slopes of the great green macaws and the blue-headed macaws were significantly steeper than those of the African grey parrots and the blue-throated macaws (GLMM: EST = − 4.457, SE = 1.220, *z* = − 3.657, *p* < 0.001). Finally, the age effect on response accuracy was not significant (GLMM: EST = 0.086, SE = 0.126, *z* = 0.683, *p* = 0.495).

### Patterns of failure

**Fig. 4:**
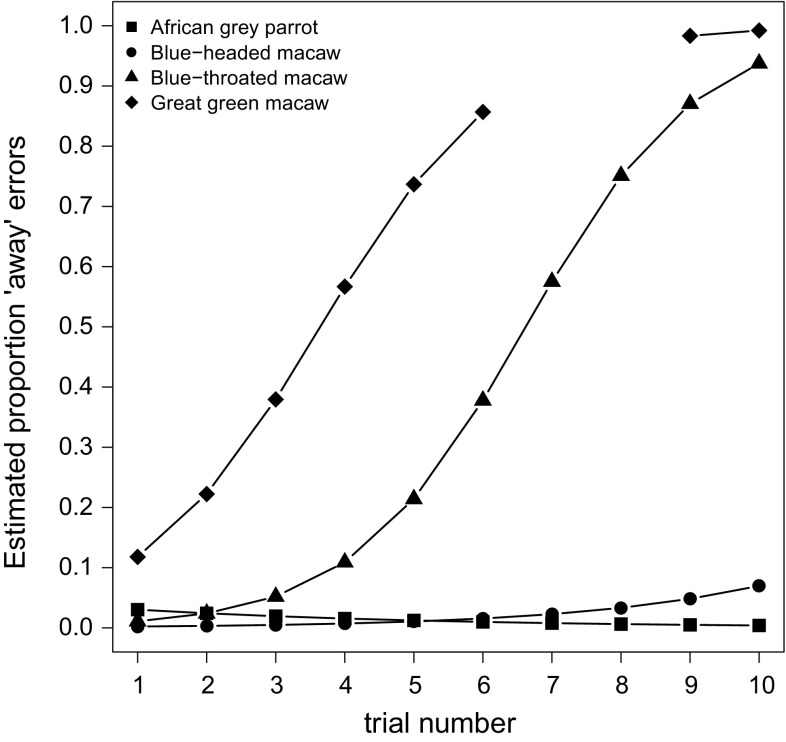
Each species’ estimated change in the proportion of ‘away’ failures (i.e., cases where the contact made with the cylinder was directed away from the food, and thus not food-related) over the 10 trials predicted from the generalized mixed model regression analysis, with error type (toward or away) as a binary outcome. The gap in the regression line for the great green macaw is due to the fact that there were no errors at the seventh and eighth trial in this species

The recalculations of the scores according to the coding criterion, where failures were coded only if the touch was directed *toward* the reward, are shown in Table 1. Averaging across all species, the recalculated scores using this new coding criterion were significantly higher than the original scores (Paired *t* test: *t* (37) = 4.307, *p* < 0.001). This increase was particularly noticeable for the blue-throated macaws and the great green macaw (*p* < 0.001 for both species); however, there was only a marginal increase for the African grey parrots (*p* = 0.900) and the blue-headed macaws (*p* = 0.451, Table 1). Analysis of response accuracy using these new scores replicated the results of our initial model, showing a significant effect of trial (GLMM: EST = 4.878, SE = 0.846, *z* = 5.768, *p* < 0.001). Additionally, the combined slopes of the great green macaws and the blue-headed macaws were again significantly steeper than those of the African grey parrots and the blue-throated macaws (GLMM: EST = − 4.616, SE = 1.563, *z* = − 2.953, *p* = 0.003). Our separate analysis of the different error types found a significant effect of trial (GLMM: EST = 4.175, SE = 1.436, *z* = 2.907, *p* = 0.004), suggesting that the proportion of errors ‘away’ from the food increased over trials (Fig. 4). Furthermore, the difference in slope between African grey parrots and blue-throated macaws was also significant (GLMM: EST = − 7.261, SE = 3.518, *z* = −2.064, *p* = 0.039). No other effects were significant, despite the seemingly large differences between the species shown in Fig. 4. One presumable reason for this is that there were too few errors during the later trials in the experiment, so the possible differences between the species could not be found reliably.

Response times ranged from 1 to 107 s, with an average of 5.26 s. The distribution of the response times was positively skewed, with a few extreme large values, notably in the blue-headed macaws. In order to reduce skewness and the effect of extreme values, a reciprocal transformation was applied (1/time) in the regression analysis. The overall trial effect was significant (LMM: EST = 0.282, SE = 0.031, *df* = 33.05, *t* = 8.963, *p* < 0.001), suggesting that all species’ responses became faster over the ten trials. In contrast to the analysis of the response accuracy, including the interaction between species and trial did not significantly improve the fit of the regression model (*χ* ^2^(3) = 2.486, *p* = 0.478), suggesting that the effect of trial on response times was comparable across the four species. Finally, the age effect was not significant (LMM: EST = − 0.012, SE = 0.009, *df* = 32.55, *t* = − 1.293, *p* = 0.205).

## Discussion

The parrots in this study performed correctly on an average of 45% of trials. This performance is markedly poorer than the average 96% success rates of *Corvus* species on the cylinder task (Kabadayi et al. 2016), despite both groups showing similar neuronal densities and brain sizes, and often showing similar performances in taxing cognitive tests (Kabadayi et al. 2016; MacLean et al. 2014; Olkowicz et al. 2016; Güntürkün and Bugnyar, 2016). The scores of the parrots in our study also align with the 50.8% performance of orange-winged amazon (*Amazona amazonica*), which was the only parrot species tested previously on the cylinder task (MacLean et al. 2014). Thus, our findings do not support the hypothesis that higher numbers of pallial neurons in birds predict better performance in cognitive tasks (Herculano-Houzel 2017). Nevertheless, as the brain is not a homogenous organ, but a network of various specialized regions, specific cognitive performances might be better explained by allometrically corrected sizes of specific brain regions, especially associative regions that support executive functions (Lefebvre and Sol, 2008). In mammals, detours around transparent barriers are mediated by prefrontal regions—the dorsolateral prefrontal cortex and the orbitofrontal cortex (Diamond 1990; Wallis et al. 2001). In birds, the caudal part of the nidopallium, the nidopallium caudolaterale (NCL), is considered analogous to the mammalian prefrontal cortex (Güntürkün, 2012) and is involved in other motor self-regulation tasks (Kalt et al. 1999). The nidopallium is similarly enlarged in parrots and corvids, (Sayol et al. 2016; Mehlhorn et al. 2010); however, it is possible that they differ when it comes to the relative size of the NCL. Similarly, it might be that the neuronal numbers in the NCL as well as connectivity among brain regions differ between corvids and parrots (Herculano-Houzel, 2017; Sherwood et al. 2008), or that other brain regions are more important in detour tasks in birds. Future research is required to better understand the functional properties of different regions in bird brains and possible differences in neuroarchitecture and function between corvids and parrots.

However, performance in the cylinder task may be influenced by many factors other than neuronal capacity and cognitive ability, and thus it may not reflect a species’ genuine motor self-regulation skills. Among others, learning processes, motivation to explore objects, or cognitive development are potential confounding factors that could affect the performance in the cylinder task. We will discuss and review them below and raise the question whether the cylinder task is always a valid test of motor self-regulation.

In the current study, we found rapid improvements in performance, strongly suggestive of learning processes, in the great green macaws and the blue-headed macaws, and although less rapidly compared to these two species, the African grey parrots and the blue-throated macaws also increased their scores across trials. Kabadayi et al. (2016) suggested that one potential cause of such learning effects and the poor performances on initial trials might be the lack of experience with transparent surfaces. However, parrots tested in our study had interacted with transparent objects such as transparent windows and Plexiglass panels in previous experiments before being tested on the cylinder task. Thus, the observed learning effect is unlikely to be attributable to the lack of experience with the transparent surfaces per se.

We also observed a reduction in response times, which is generally regarded as a sign of learning (Koopmans et al. 2003). Individuals of all species became quicker in retrieving the reward over trials, regardless of whether they first touched the cylinder surface or not. Another cylinder task study on song sparrows (*Melospiza melodia*) showed that the subjects reached perfect performance after around 50 trials (Boogert et al. 2011). It is possible that having more than 10 trials would have notably increased the accuracy in all four parrot species. In fact, the scores of the great green macaws reached almost perfect, and stable, accuracy already around trial six and stayed there until the end of the experiment.

This type of improvement may have broader implications for how motor self-regulation is measured. A relatively rapid improvement over trials in the cylinder task and a subsequent ceiling level of perfect accuracy found by ourselves and previous studies (Vernouillet et al. 2016; Boogert et al. 2011) deviate from the pattern seen in other types of motor inhibition tasks, where improvements are unusual (Cohen and Poldrack, 2008; Berkman et al. 2014). In contrast to the cylinder task, classical motor inhibition tasks leave little opportunity for learning to occur, because of their task-switching component: On certain trials, the subjects must refrain from the previously reinforced responses and instead choose a different response. This might explain why perfect accuracy did not occur in a study on squirrel monkeys (*Samiri sciureus*) when a task-switching component was added to a detour-reaching task around transparent barrier (Parker et al. 2012). A study on common marmosets (*Callithrix jacchus*) also suggested that detour tasks around transparent barriers might not always measure inhibition, as it was found that depletion of serotonin in the prefrontal cortex impairs detour-reaching behavior around the barrier *during* the task acquisition, but not after the task is learned (Walker et al. 2006). This suggests that the cylinder task only measures motor self-regulation before there is a detectable overall improvement, which makes the commonly used score of performance across trials difficult to implement, especially in a comparative context. There is a risk that one compares learning speed rather than inhibition.

Yet another possible learning effect in the cylinder task paradigm may apply and substantially influence the results, namely the ability to learn to transfer from the opaque cylinder to the transparent one. In the current study, as well as in those we replicated, the subjects received relatively few training trials on the opaque cylinder. Even if the animal readily learned to retrieve the reward from the opaque cylinder, it is possible that more training still would have been required to entrench the affordances of the task sufficiently for an immediate transfer to the transparent cylinder to occur. A study testing common marmosets on a detour task around a transparent barrier found that success was determined by a combination of inhibitory skill and the ability to transfer the detour response from an opaque to a transparent barrier, where both skills are mediated by different brain regions (Wallis et al. 2001). Thus, species differences might not only reflect differences in motor self-regulation and rule learning speed, but also the ability to transfer between the two types of cylinders. For example, corvids of the genus *Corvus*, along with great apes, perform close to perfectly (Kabadayi et al. 2016; MacLean et al. 2014), but this could equally be a result of good transfer skills or inhibitory capacity, or a combination thereof. Recently, it was suggested that the total number of pallial neurons might be a better predictor of transfer ability and learning speed, rather than of general cognitive skills (Güntürkün et al. 2017). However, as parrots have similar number of pallial neurons to corvids, this should not explain the differences, unless—as stated before—there are other neuroanatomical differences. In any case, future comparative studies using the cylinder task should take into account the task transfer skills of the species in order to avoid confounding effects.

Ontogeny is another parameter that may influence cylinder task performance. Children undergo a developmental period, between 6 to 12 months of age, where they have difficulties in retrieving objects from behind transparent barriers (Diamond 1990). Similarly, rhesus monkey infants gradually improve in the same tasks between 1 and 4 months (Diamond 1990). In our study, we did not find an overall age effect across species, but since all African grey parrots and blue-headed macaws were juveniles around 1 year old (and showed the poorest performance in the task), they might not have fully developed their inhibitory skills. Future comparative studies should pay attention to overall differences in cognitive ontogeny in different species and specifically test how development affects the detour response across different species.

A species’ object exploration style is a motivational aspect that can influence performance. The cylinder task can generate false negatives if the tested animals touch the cylinder in order to explore the surface rather than in an attempt to reach for the reward, which might happen especially since touching the cylinder does not infer any major cost. Considering that touches not directed toward the reward behind the barrier are unlikely to be inhibition failures (Noland and Rodrigues 2012), we ran additional analyses on the failure patterns. They revealed that most failures by the African grey parrots and the blue-headed macaws appeared to be attempts to reach directly for the reward, thus representing true errors, whereas the great green macaws and blue-throated macaws also frequently touched the cylinder in a manner that did not seem to be food-directed. Interestingly, the frequency of such non-food-related failures increased across trials for the great green macaws and blue-throated macaws constituting the majority of their failures in later trials. Indeed, all failures of the great green macaws in the last five trials were of this nature, so it is unlikely that the failing individuals did not know the correct solution of the task. Instead those failures might have occurred due to exploration or boredom resulting from an exposure to repeated trials requiring an identical response. Kabadayi et al. (2016) reported similar failure patterns for New Caledonians and jackdaws, where the individuals touched the barrier likely in an attempt to explore the surface rather than to reach the reward. Such examination of the failure patterns was missing in the large-scale study (MacLean et al. 2014), and neglecting these analyses might have underestimated the scores of some species.

It is worth noting that focusing only on the species average scores might miss remarkable individual performances. For example, although the blue-throated macaw’s average score was low, there were two individuals successful on all trials (Fig. 2). Such individual variation has big implications in the interpretation of large comparative datasets, especially with small sample sizes of each species (Thornton and Lukas, 2012).

In summary, we found that the four parrot species performed relatively poorly in the cylinder task despite having large brains and high pallial neuronal densities, and despite other species from this order demonstrating well-developed cognitive skills in other domains, including self-control (Güntürkün and Bugnyar 2016). This suggests two possibilities: (1) Neither brain volume nor the number of neurons in the pallium is a good predictor of the cylinder task performance within birds, or at least within Psittaciformes. Instead, the relative size, the number of neurons or other anatomical features of the specific brain regions might play an important role. (2) The cylinder task may not be an adequate test to capture motor self-regulation skills in parrots (and some other animals). To better tease these two factors apart, further comparative studies are needed, which ideally include a battery of different tests on motor inhibition and that specifically examine factors that may influence performance in this and other motor inhibition tasks.

## Electronic supplementary material

Below is the link to the electronic supplementary material.

Supplementary material 1 (MOV 33374 kb)
